# ^18^F-fluorothymidine PET imaging in gliomas: an update

**DOI:** 10.1007/s12149-017-1183-2

**Published:** 2017-06-13

**Authors:** Alexandra Nikaki, George Angelidis, Roxani Efthimiadou, Ioannis Tsougos, Varvara Valotassiou, Konstantinos Fountas, Vasileios Prasopoulos, Panagiotis Georgoulias

**Affiliations:** 1 Department of Clinical Physiology, KHSHP, 20 Ahvenistontie Str., 13530 Hämeenlinna, Finland; 2 Department of Nuclear Medicine, University Hospital of Larissa, Mezourlo, 41110 Larissa, Greece; 3 PET/CT Department, Hygeia Hospital, 4 Erythrou Stavrou Str., 15123 Athens, Greece; 4 Department of Neurosurgery, University Hospital of Larissa, Mezourlo, 41110 Larissa, Greece; 5 Department of Nuclear Medicine, Hygeia Hospital, 4 Erythrou Stavrou Str., 15123 Athens, Greece

**Keywords:** Fluorothymidine, Glioma, MRI, PET, Prognosis

## Abstract

Brain neoplasms constitute a group of tumors with discrete differentiation grades, and therefore, course of disease and prognosis. Magnetic resonance imaging (MRI) remains the gold standard method for the investigation of central nervous system tumors. However, MRI suffers certain limitations, especially if radiation therapy or chemotherapy has been previously applied. On the other hand, given the development of newer radiopharmaceuticals, positron emission tomography (PET) aims to a better investigation of brain tumors, assisting in the clinical management of the patients. In the present review, the potential contribution of radiolabeled fluorothymidine (FLT) imaging for the evaluation of brain tumors will be discussed. In particular, we will present the role of FLT-PET imaging in the depiction of well and poorly differentiated lesions, the assessment of patient prognosis and treatment response, and the recognition of disease recurrence. Moreover, related semi-quantitative and kinetic parameters will be discussed.

## Introduction

Brain neoplasms can be classified into two main groups, primary tumors and metastatic brain lesions which are more common. Gliomas represent the most frequent type of primary brain tumors and mainly consist of malignant neoplasms; more than half of these lesions are glioblastomas [1]. The incidence of malignant gliomas is approximately 3–5/100,000 cases, with slightly higher incidence in males and a peak at the sixth decade of life [1, 2]. They arise from the glial cells and constitute a heterogeneous group of neoplasms characterized by different cell origin and developmental pattern [3]. According to their malignant potential, they are categorized into four grades (I–IV). Grade I and II lesions correspond to non-invasive gliomas, whereas grades III and IV include invasive tumors with worse outcome and poorer prognosis [3, 4]. However, there may be overlaps among the morphological and diagnostic characteristics used for grading purposes. Moreover, genetic/epigenetic evidence is taken into account for the determination of prognosis and in the therapeutic decision-making, including signaling pathways and molecular markers such as mitotic marker MIB-1, isocitrate dehydrogenase 1 (IDH1) mutations, 1p/19q loss for oligodendrogliomas, epigenetic silencing of methylguaninmethyltransferase (MGMT) gene promoter, epidermal growth factor receptor (EGFR) amplification, and microRNAs [2, 4–9]. In general, current treatment management includes surgical excision of the tumor, radiation treatment and chemotherapy with alkylating factors (temozolomide).

Magnetic resonance imaging (MRI) with gadolinium (Gd)—enhancement is the method of choice for the initial diagnostic investigation of brain lesions, as well as for the evaluation of treatment response and the early depiction of disease recurrence [10]. ^18^F-fluorodeoxyglucose (^18^F-FDG)—positron emission tomography (PET), the conventional PET imaging technique in oncology, has been also used in patients with brain lesions, either at their presentation, or for the assessment of response to treatment and detection of recurrence [11, 12]. Notably, despite its applicability, ^18^F-FDG is not considered as the most appropriate radiotracer for the investigation of brain lesions due to the high background activity. For this reason, several radiopharmaceuticals have been developed in this field, such as radiolabeled amino acids, ^18^F-choline, hypoxia detection agents and tumor proliferation markers (Table 1).

**Table 1:** Classification of positron emission tomography radiotracers for glioma investigation based on molecular processes under study

Molecular processes	Radiotracers	
Glucose metabolism	2-[^18^F] fluoro-2-deoxy-d-glucose	^18^F-FDG
Membrane biosynthesis	^11^C-Choline	
	^18^F-Fluorocholine	
Oxygen metabolism	^18^F-Fluoromisonidazole	^18^F-FMISO
Amino acid transport, protein synthesis	^11^C-Methionine	^11^C-MET
	^18^F-Fluoroethyltyrosine	^18^F-FET
	^18^F-Fluorotyrosine	^18^F-TYR
	^18^F-Fluoromethyltyrosine	^18^F-FMT
	^18^F-Fluorodopa	^18^F-DOPA
Proliferation rate	^18^F-Fluorothymidine	^18^F-FLT

## Radiolabeled analog of fluorothymidine (^18^F-FLT)

^18^F-FLT is a radiolabeled analog described initially as a selective inhibitor of DNA synthesis [13]. It was introduced by Wilson et al., and in an alternative form, by Shields and Grierson [14, 15]. Given that thymidine is a nucleoside encountered only in DNA, the radiolabeled analog was proposed to reflect tissue proliferation rate [16]. ^18^F-FDG enters cells by active transport through nucleoside transporters (salvage thymidine pathway), as well as by passive diffusion [17]. However, it does not incorporate into the DNA chains and remains trapped after phosphorylation by thymidine kinase-1 (TK-1), which is increased at the S-phase of the cell cycle, reflecting, in this context, tumor proliferation [16–18].

In brain tumors, newer evidence suggests that ^18^F-FLT uptake depends mainly on the increased permeability, intracellular transport and influx after the disruption of the blood–brain barrier (BBB) or the function of nucleoside transporters in case of intact BBB, whereas the contribution of the metabolic trapping through phosphorylation seems to be less important [19, 20]. These findings became available using kinetic analysis in animal models and human series, both in newly diagnosed gliomas and lesions after treatment. Moreover, it is now recognized that the major limiting factor of ^18^F-FLT uptake is the transport mechanism, while its accumulation is mainly attributed to the transport and influx rate [21, 22]. Interestingly, an association has been demonstrated in grade III and IV gliomas between ^18^F-FLT uptake and the metabolic rate (as described by K3 constant), indicating—at least in part—the contribution of the metabolic factor in radiotracers’ intracellular maintenance in this subgroup of tumors [22]. Further, in recurrent lesions, a link has been suggested between radiotracer uptake and the combined influence of influx and metabolic rate (as described by Ki constant) [23]. Consequently, it remains unclear whether ^18^F-FLT could actually demonstrate cell proliferation in brain tumors. There is also a possibility to reflect only BBB disruption, a characteristic that could lead to controversy regarding radiotracer specificity in tumor detection. In particular, the non-specific binding of the radiotracer may be related to the false positive results, and could impair proper tumor delineation and characterization.

## Tumor detection and grading

**Fig. 1:**
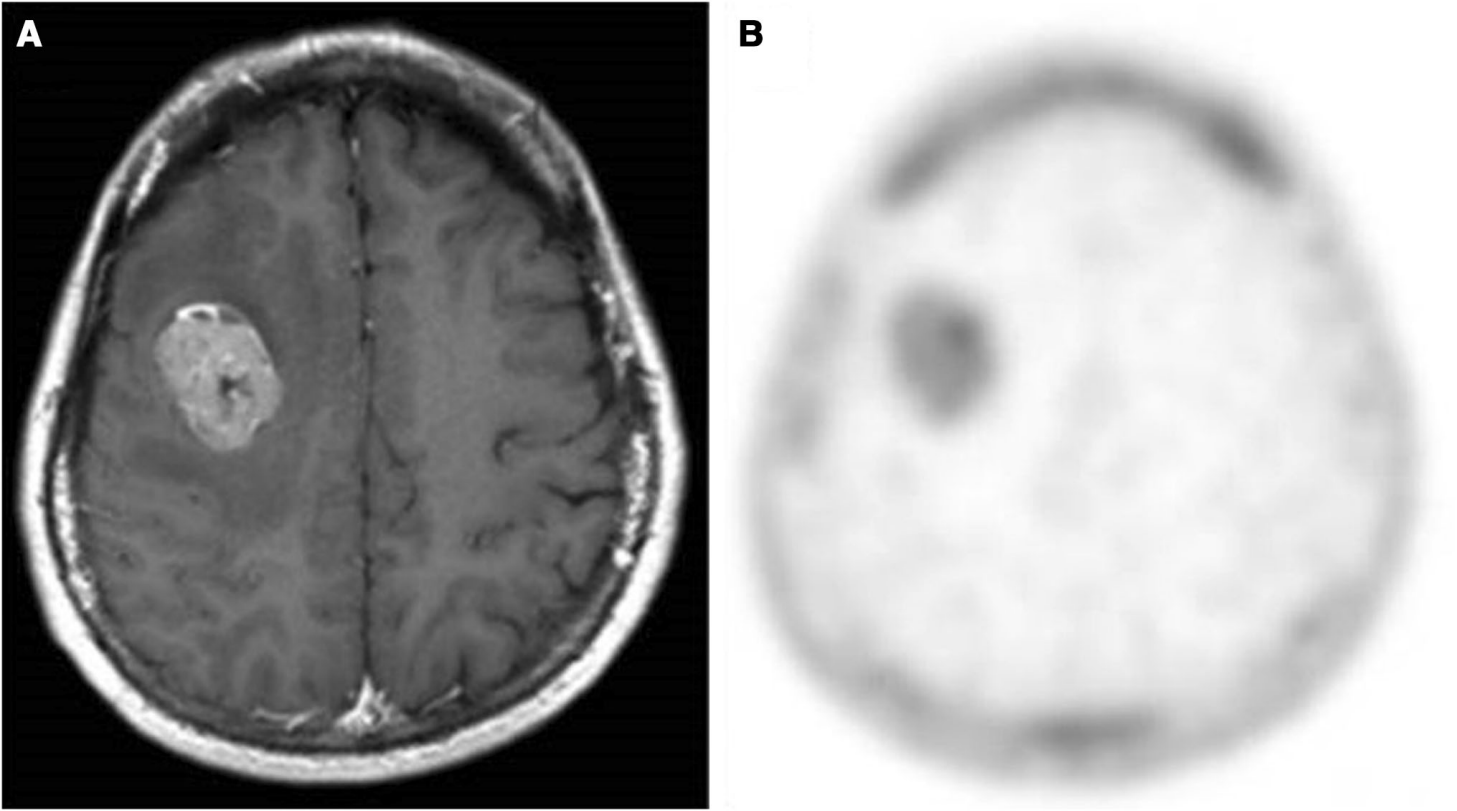
Newly diagnosed grade IV glioblastoma. **a** Magnetic resonance image with contrast enhancement, **b** ^18^F-FLT PET image (tumor-to-normal ratio 11.67). This research was originally published in [26] © by the Society of Nuclear Medicine and Molecular Imaging, Inc. *FLT fluorothymidine

Accurate tumor detection and delineation, as well as grading before surgical resection, are of great importance. These parameters have influences on the surgical procedure, the post-surgical treatment management and the patient prognosis. In addition, it is crucial to identify tumor grade in inoperable cases. Figure 1 shows a grade IV glioblastoma as depicted by MRI (A) and ^18^F-FLT (B) techniques.

^18^F-FLT imaging has been reported to depict accurately the biopsy site when glioma is suspected [24]. The sensitivity of the method in detecting high-grade gliomas could reach 100%; however, overall sensitivity is lower (~83%) due to significant differences in radiotracer uptake between high- and low-grade tumors [12, 25, 26]. Moreover, the sensitivity and accuracy of the technique in differentiating high- vs. low-grade tumors is ~92% [27]. In high-grade lesions, its ability to discriminate between grade III vs. grade IV gliomas is also high, but it is lower when differentiating grade II vs. grade III tumors [25, 26]. In general, ^18^F-FLT imaging is considered less valuable for low-grade gliomas, as they present no or little radiotracer uptake [28]. Nevertheless, this characteristic can be used in tumor grading. Compared to advanced MR techniques and spectroscopy, ^18^F-FLT imaging can provide important evidence in the discrimination of tumors between grades II and IV and grades III and IV, despite its lower performance for grade III vs. grade IV differentiation [29].

In accordance with ^11^C-methionine (^11^C-MET) imaging, the use of ^18^F-FLT leads to larger volume when delineating the tumor, compared to Gd-enhanced MRI method. Possibly, radiotracer accumulation may precede substantial BBB breakdown, or the radiotracer could be a more sensitive marker of BBB breakdown. Nevertheless, previous reports suggested a fairly good agreement in tumor volume definition between MRI and PET studies. The two diagnostic methods contribute supplementarily to the delineation of the tumor burden, even though tumor margins may be defined less accurately based on FLT uptake [22, 30, 31].

Primary or progressive tumors with no or little enhancement in MRI images were found not to concentrate ^18^F-FLT, as expected based on the reported strong association between ^18^F-FLT uptake and Gd- enhancement [20, 32]. Typically, grade II gliomas do not show Gd-enhancement, ^18^F-FLT uptake or modifications in cerebral blood volume (CBV) maps. On the other hand, grade III gliomas usually demonstrate mild Gd-enhancement and radiotracer uptake, whereas grade IV gliomas show high Gd-enhancement and ^18^F-FLT uptake. However, a grade III glioma may demonstrate MRI and magnetic resonance spectroscopy (MRS) characteristics of glioblastoma multiform (including apparent diffusion coefficient (ADC)), despite the presence of mild ^18^F-FLT uptake, or a grade IV lesion may present non-profound perfusion changes though high radiotracer uptake [29]. Moreover, intratumoral heterogeneity could be apparent in gliomas, and the related measurements may provide additional information about tumor characteristics [33]. Finally, in a rat and a mouse model, ^18^F-FLT uptake has been related to tumor development, growth and size [34, 35].

Notably, the standardized uptake value (SUV) calculations alone may not be adequate to assess the actual proliferative cellular activity. Uptake results could be also associated with non-specific leakage, probably representing BBB breakdown in high-grade gliomas. Therefore, the evaluation of compartmental-derived kinetic parameters has a significant role in this field [36]. ^18^F-FLT concentration remains constantly low in normal brain tissue allowing high tumor-to-background ratio, although SUV values may be low [37]. Chen et al. reported that gliomas concentrate radiotracer rapidly, reaching the maximum uptake after 5–10 min and remaining stable for about 75 min [37]. Apparently, kinetic analysis can vary among studies in one or more of the following points: differences in the compartmental model, corrections for metabolites, the K constants (representing intracellular transport and metabolism of ^18^F-FLT), as well as in methods used to discriminate vascular and tissue activity. For high-grade lesions, no substantial differences have been observed between blood measurements from arterial blood sampling and PET derived venous measurements, indicating that such calculations can be easily applied in the clinical setting [38]. Regarding the circulating metabolites due to FLT metabolism, even a limited set of blood samples is adequate in the kinetic analysis of radiotracer retention, and for metabolic rate calculations [39]. In general, radiotracer kinetics can provide useful information about the tumor characteristics and patient prognosis, leading to a better therapeutic management compared to the semi-quantitative results alone [36–38]. However, ^18^F-FLT PET findings in previously untreated low-grade gliomas were correlated with overall survival, not event-free survival, possibly due to referral bias [40].

## ^18^F-FLT correlations with biomarkers

Given its association with TK-1 at the salvage DNA synthesis pathway, ^18^F-FLT was proposed to be correlated with proliferation markers. After analyzing the results of previous studies with a total sample of 509 patients, Chalkidou et al. reported that ^18^F-FLT kinetic parameters and SUV_max_ values were associated with Ki-67 measurements [41]. Moreover, higher reproducibility was achieved when mean instead of maximum SUV values were analyzed, as well as when surgically excised sections (not biopsy samples) were used [41]. In comparison to ^11^C-MET, the association of ^18^F-FLT uptake with Ki-67 was found to be more significant, whereas the highest Ki-67 percentage glioblastoma cases exhibited high ^18^F-FLT, but moderate ^11^C-MET, uptake [25]. Further, ^18^F-FLT uptake was significantly associated with Ki-67 both in newly diagnosed and recurrent brain tumors; however, the correlation in recurrent lesions was weaker [26]. In a rat glioblastoma model, a good agreement was confirmed between ^18^F-FLT uptake and Ki-67 staining in both bevacizumab-treated and non-treated groups, suggesting an association between radiotracer uptake and angiogenesis inhibition [34]. Moreover, diminished proliferation rate (as assessed by Ki-67), increased cell death and diminished ^18^F-FLT uptake were observed after irradiation of glioblastoma cells in a mouse model [35]. Consequently, ^18^F-FLT PET imaging may provide additional information regarding tumor cell proliferation in radiation-treated areas. Finally, normal-to-background ratio in ^18^F-FLT imaging of newly diagnosed and recurrent tumors was positively correlated to the expression of a 58-kD microspherule protein highly produced in grade IV gliomas [36]. Furthermore, both radiotracer uptake and the expression of the above mentioned protein were linked to Ki-67 expression and overall survival in newly diagnosed lesions, implying the potential role of these parameters as targets for proliferation therapy, as well as in therapy assessment [42].

## Evaluation of recurrence—residual disease

Pseudo-progression and pseudo-regression may present about two months after radiation therapy and temozolomide administration in patients with gliomas. Pseudo-progression refers to increased Gd-enhancement in MRI images despite response to treatment, whereas pseudo-regression corresponds to cases characterized by tumor progression despite decreased Gd-enhancement. Therefore, in the clinical setting, the main question is whether patient symptoms could be attributed to either recurrent disease or radiation necrosis [43].

**Fig. 2:**
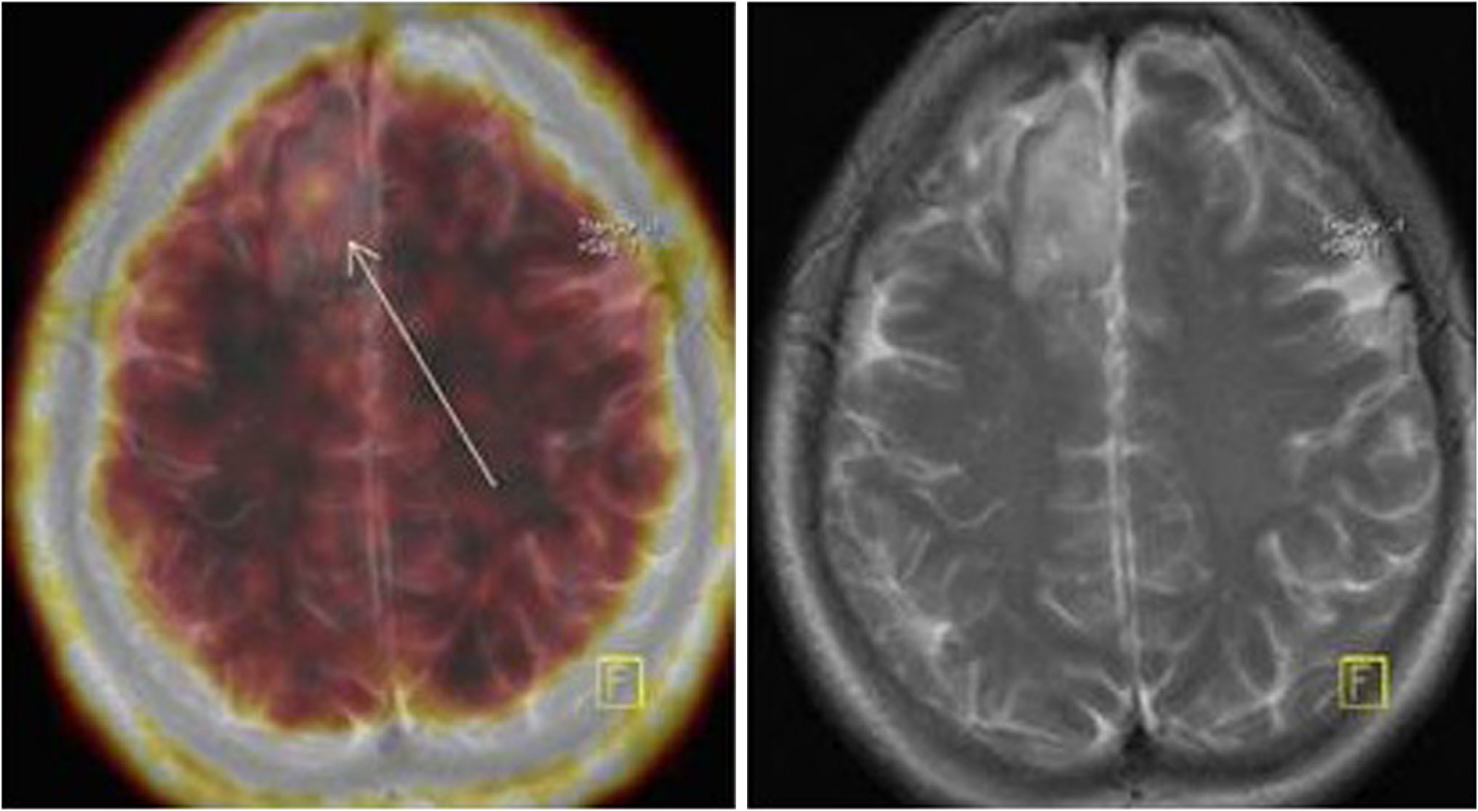
Grade II oligodendroglioma recurrence (*arrow*), low ^18^F-FLT uptake (SUV_max_ 0.85, tumor-to-background ratio 7.0). *FLT fluorothymidine, *SUV* standardized uptake value

Studies not only in cell lines and animal models but also in humans have been performed investigating the role of semi-quantitative and dynamic kinetic ^18^F-FLT parameters in disease recurrence and treatment response. Since ^18^F-FLT uptake is mostly attributed to BBB disruption, imaging findings can be associated with necrosis after radiation therapy, or the presence of proliferating tissue [20]. Furthermore, unspecific radiotracer uptake may lead to false positive results [18]. Figure 2 shows a grade III cerebral tumor recurrence.

**Fig. 3:**
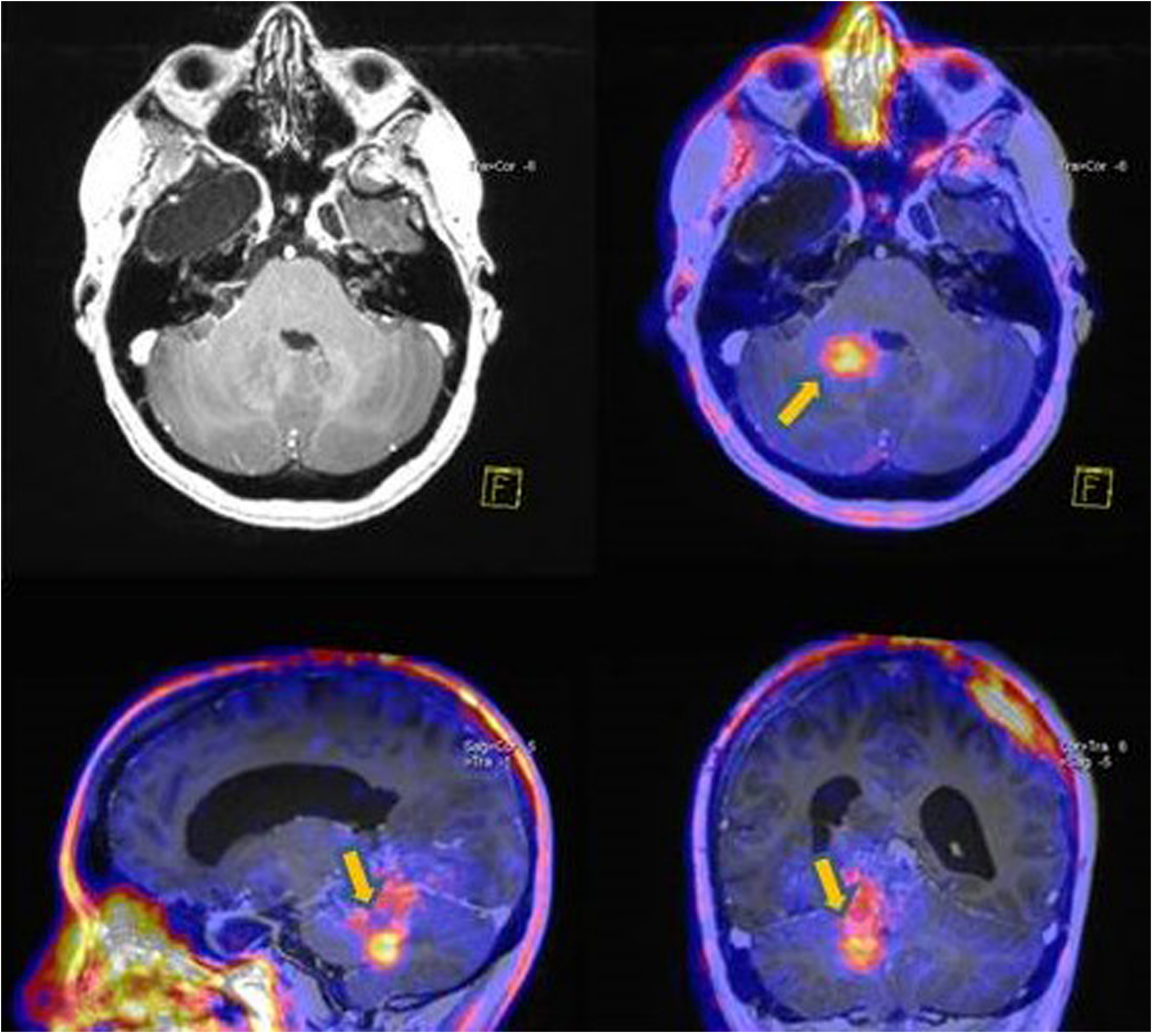
Anaplastic oligoastrocytoma grade III recurrence. Increased uptake of FLT at the right cerebral hemisphere (SUV_max_ 1.22, tumor-to-background ratio 4.8). Fusion of PET and MRI images. *FLT fluorothymidine, *MRI* magnetic resonance imaging, *PET* positron emission tomography, *SUV* standardized uptake value

In comparison to ^18^F-FDG imaging, ^18^F-FLT was found to have higher sensitivity and accuracy in the investigation of disease recurrence, despite similar specificity [44]. Moreover, ^18^F-FLT technique has lower ability to distinguish recurrent lesions according to their grade, than newly diagnosed tumors [26, 45]. In particular, using SUV_max_ as a quantitative parameter in the differential diagnosis between radiation necrosis and disease recurrence, ^18^F-FLT technique yielded high sensitivity but moderate specificity, limiting its use as only a supplementary tool in this field. Although treatment-induced changes could be differentiated from recurrent lesions based on radiotracer uptake, as well as high- vs. low-grade recurrent lesions, the accumulation of ^18^F-FLT in low-grade gliomas is low (Figs. 2, 3). For this reason, ^18^F-FLT imaging should not be used in low-grade recurrent brain tumors [28].

Nevertheless, there is evidence that no ^18^F-FLT uptake in MRI enhanced lesions could actually reflect the absence of recurrence [46]. Notably, tumor-to-background ratio may be more accurate index in the discrimination between necrotic vs. malignant tissue, compared to SUV_max_ [47]. Further, in residual tumor delineation, ^18^F-FLT-defined tumor volume may differ from T2-defined tumor volume and contrast-enhanced regions, possibly resulting in modifications in radiation tumor targets (including plan tumor volume and boost tumor volume) [48]. Consequently, molecular information obtained through ^18^F-FLT imaging may be useful in radiation treatment planning, both for dose escalation in residual cancer cells and the protection of the surrounding normal tissue.

Finally, ^18^F-FLT kinetic parameters were reported to perform better than semi-quantitative measurements in the differentiation between radiation necrosis and recurrent disease [49]. Particularly, Enslow et al. demonstrated the value of Ki_max_ in tumor ^18^F-FLT kinetics assessment, compared to necrotic tissue characteristics due to radiation [50]. On the other hand, no significant difference was observed between necrotic vs. malignant tissue, with regard to SUV_max_ parameter [50].

## Treatment response: yielding prognostic information

Depending on the location and differentiation state of each lesion, the therapeutic management of gliomas may include surgery, radiation therapy and/or chemotherapy. Subsequently, the evaluation of treatment response is of great importance since it is directly related to survival. Contrast enhancement MRI, with additional T2 and FLAIR-weighted MRI response assessment after chemotherapy, is the method of choice to evaluate treatment response, whereas MRS and radiolabeled amino acids PET imaging may be also helpful [1]. Regarding ^18^F-FLT technique, several uptake and kinetic parameters (such as SUV_max_, tumor-to-background ratio and proliferative volume—the volume of the proliferation section of the tumor as described by ^18^F-FLT PET) have been investigated for the evaluation of treatment response and their ability to provide prognostic information. In a mouse model, the influence of radiation on the pattern and degree of radiotracer uptake was studied, showing important associations with micro-environmental changes in glioblastoma tumors [35]. Irradiated lesions appeared with a lower and more uniform uptake pattern, whereas non-irradiated lesions exhibited peripheral uptake with a photopenic center. Therefore, ^18^F-FLT PET imaging may contribute to radiation therapy response assessment; however, clinical trials in this area are lacking [51].

^18^F-FLT technique has been demonstrated to provide useful information for the evaluation of response to chemotherapy, yielding prognostic value in newly diagnosed high-grade gliomas and recurrent brain tumors. After enrolling 21 patients with recurrent tumors treated with irinotecan-bevacizumab, Chen et al. demonstrated the predictive capability of the technique in depicting responders vs. non-responders and its correlation with progression-free and overall survival [52]. These findings were also confirmed in subsequent studies [53, 54]. Interestingly, in a study sample of 30 patients treated with bevacizumab, radiotracer uptake changes at 6th week post-treatment initiation were found to be the strongest independent predictor of survival [54].

In everyday clinical practice, SUV values may be the only semi-quantitative measurements recorded [23]. Notably, in previous studies, conflicting prognostic information was obtained based on SUV_max_ measurements. However, these discrepancies may be related to sample differences among studies. On the other hand, tumor-to-background measurements seemed to be a more reliable prognostic indicator [55, 56]. Moreover, Idema et al. enrolled glioma patients with either untreated or recurrent lesions and found that ^18^F-FLT-derived proliferative volume was significantly correlated to overall survival [17]. In comparison to Gd-enhanced MRI method, radiotracer uptake may correspond to larger areas. However, these areas were found to be related to relative cerebral blood volume (rCBV) with higher accuracy [17]. Further, proliferative uptake volumes were associated with overall survival in patients with recurrent gliomas, and the observed association was stronger than that using MRI-derived volume [56].

Differences in ^18^F-FLT kinetic parameters in patients with gliomas can be attributed to either tumor-induced consequences or changes related to treatment response; therefore, kinetic analysis may provide important prognostic information [23, 57]. According to Wardak et al., ^18^F-FLT kinetics may be useful in discriminating long-term vs. short-term survivors with high diagnostic accuracy, possibly leading to a more individualized therapeutic management [55]. Moreover, evidence obtained through ^18^F-FLT kinetic analysis was reported to predict overall survival more accurately in comparison to ^18^F-fluorodopa kinetics [58]. Consequently, if ^18^F-FLT kinetic data are obtained early in the treatment of recurrent brain tumors with bevacizumab and irinotecan, useful prognostic information can be obtained with reasonable confidence [58].

## Comparison between the available imaging techniques

**Fig. 4:**
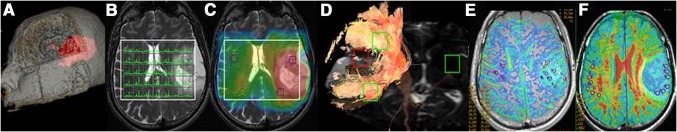
Advanced magnetic resonance imaging techniques. **a** 3D rendering, **b** 2D magnetic resonance spectroscopy, **c** metabolite mapping, **d** fiber tractography, **e** dynamic susceptibility contrast imaging, **f** diffusion imaging

MRI can offer morphological evidence in glioma patients. However, its value is limited for the evaluation of more specific information regarding the biological characteristics of the lesions. Moreover, MRI has certain limitations, particularly for the initial evaluation of tumor aggressiveness. False results have been also described, especially after radiotherapy or chemotherapy. Although advanced MRI techniques (Fig. 4) may contribute to the minimization of MRI pitfalls, certain limitations still exist, especially regarding the magnetic field inhomogeneity of the area under investigation [10]. PET imaging may contribute to the individualization of therapeutic management. Several molecular processes can be visualized depending on the radiotracer used (Table 1). Maximal or mean SUV is commonly used for the semi-quantitative evaluation of the radioactivity in the target.

^18^F-FDG PET was initially proposed due to the increased glucose metabolism in high-grade gliomas, as well as the positive association between glycolysis rate and malignancy [59]. ^18^F-FDG uptake was linked to tumor grading, showing prognostic value [60]. However, the utility of ^18^F-FDG imaging is hampered by the high glucose metabolism in normal brain areas; both the sensitivity for tumor detection and specificity for tumor delineation are significantly limited [60]. Particularly, low-grade gliomas are characterized by modest radiotracer uptake which is similar to that of white matter, and decreased uptake in comparison to gray matter [12]. Moreover, ^18^F-FDG accumulation in inflammatory tissue makes the distinction between malignancy and inflammation often challenging. Regarding stereotactic biopsy target selection, ^18^F-FDG imaging was reported to be superior compared to MRI, despite its limited value in low-grade gliomas [61].

Since cell proliferation can be related to higher metabolism of cell membrane components, radiolabeled choline was proposed for the assessment of brain lesions, particularly oligodendroglial tumors [60]. Moreover, the choline analog ^18^F-fluorocholine was considered to discriminate high-grade gliomas, metastatic lesions, and benign tumors. A main disadvantage is the high radiotracer accumulation in the choroid plexus, venous sinuses, and pituitary gland, limiting the value of the technique in the vicinity of these structures [62].

Rapid tumor growth is associated with lower oxygen levels in parts of the lesion compared to the surrounding normal tissue, while hypoxia is linked to tumor progression and resistance to radiotherapy. Uptake of nitroimidazole derivative ^18^F-fluoromisonidazole, a marker of hypoxia, was observed in high grade but not in low-grade gliomas [60]. However, this technique was associated with suboptimal imaging properties, including low target-to-background ratio and slow tumor uptake.

Increased cell proliferation in gliomas leads to higher amino acid utilization [63]. High-contrast images can be obtained using radiolabeled amino acids in both low- and high-grade gliomas, given the low normal tissue uptake. On the other hand, increased uptake due to BBB damage may be misinterpreted, and differences in amino acid transport characteristics could result in significant uptake variability [60]. Radiolabeled amino acids can contribute to the diagnosis of gliomas, while accuracy in biopsy planning may be significantly increased through the implementation of combined ^18^F-FET PET and MRI [64]. Further, radiolabeled amino acid PET may provide useful information in surgery and radiotherapy planning [31, 65]. Finally, since these radiotracers are not taken up by glycolytic inflammatory cells, a more accurate discrimination between disease progression (or recurrence) and therapy-related effects can be achieved [60].

**Fig. 5:**
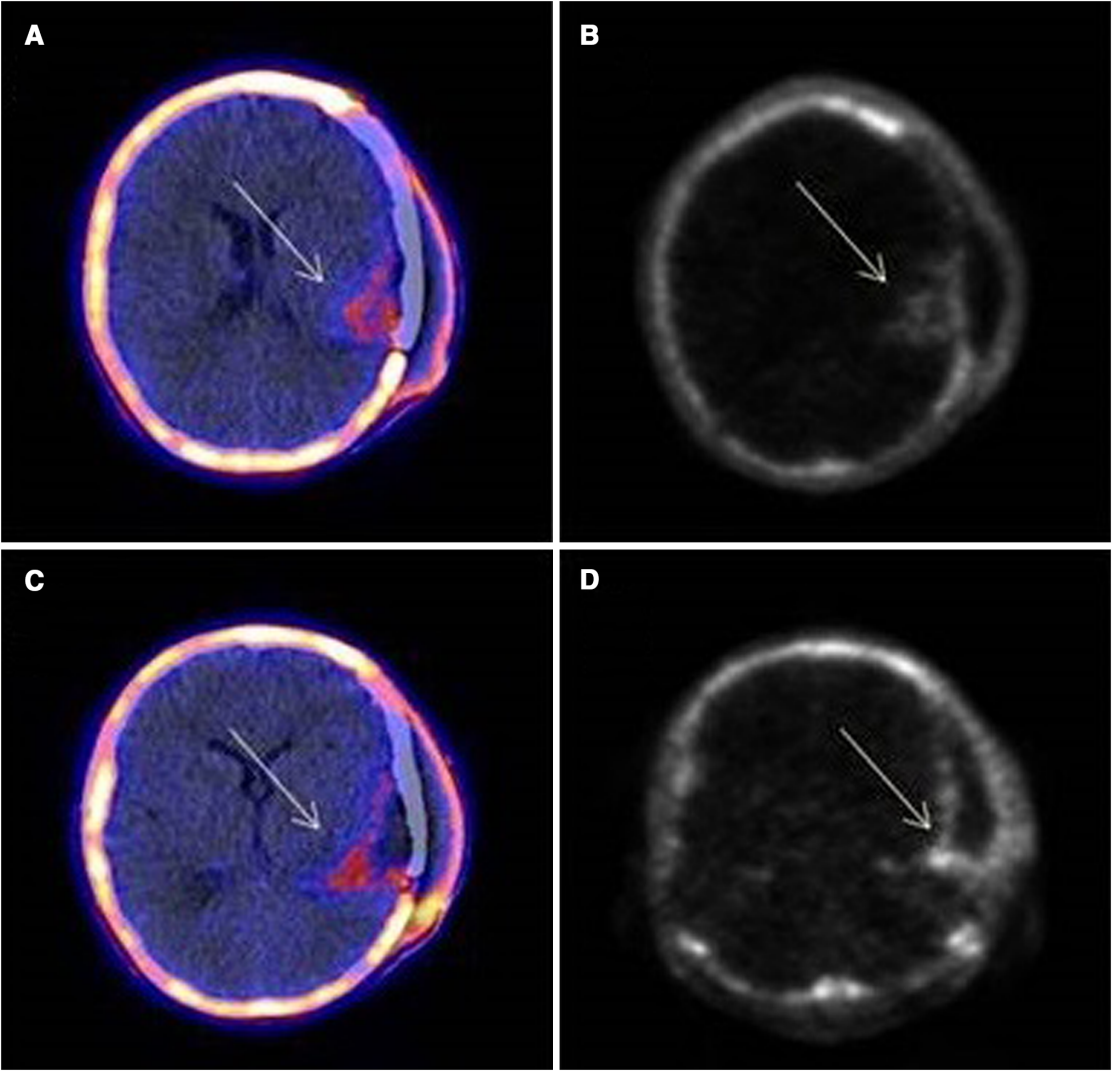
PET/CT (**a**, **c**) and PET (**b**, **d**) images of glioblastoma multiforme: increased ^18^F-FLT uptake (*arrows*) at parts of the borders of surgical excision (SUV_max_ 1.29, tumor-to-background ratio 10.75). *CT computed tomography, *FLT* fluorothymidine, *PET* positron emission tomography, *SUV* standardized uptake value

^18^F-FLT imaging focuses on the increased DNA replication observed in malignant transformation. Radiotracer uptake is lower in most regions because of the limited neuronal cell division [66]. ^18^F-FLT PET imaging can depict high-grade gliomas and assist in the discrimination between high- vs. low-grade lesions. Although this technique has been reported to depict the biopsy site, it cannot accurately identify tumor margins [24, 31]. On the other hand, ^18^F-FLT imaging may assist in the investigation of recurrence after surgical excision (Fig. 5). Finally, since structural abnormalities occur after changes in cellular proliferation, ^18^F-FLT uptake during treatment can provide valuable prognostic evidence, as well as information about treatment response.

Kinetic analysis can be performed complementing the basic ^18^F-FLT study. Notably, since cellular ^18^F-FLT uptake is limited by transport across the BBB, a complete kinetic model of radiotracer uptake, transport and metabolism could significantly improve DNA synthesis quantification. Furthermore, kinetic modeling may also provide valuable evidence for the discrimination between recurrence and radiation necrosis.

## Conclusions

Radiolabeled FLT can serve as an in vivo marker of cell proliferation, providing valuable information regarding brain malignancies in combination with tumor proliferative biomarkers. However, further prospective cohort studies, with greater number of participants, are required before ^18^F-FLT PET imaging would gain its final position in the diagnostic evaluation and prognostication of glioma patients.
